# DarNERcorp: An annotated named entity recognition dataset in the Moroccan dialect

**DOI:** 10.1016/j.dib.2023.109234

**Published:** 2023-05-12

**Authors:** Hanane Nour Moussa, Asmaa Mourhir

**Affiliations:** School of Science and Engineering, Al Akhawayn University in Ifrane, P.O. Box 104, Hassan II Avenue, Ifrane 53000, Morocco

**Keywords:** Natural language processing, Text mining, Named entity recognition, Dialectal Arabic, Corpus, BIO

## Abstract

DarNERcorp is a manually annotated named entity recognition (NER) dataset in the Moroccan dialect, also called Darija. The dataset consists of 65,905 tokens and their corresponding tags according to BIO scheme. 13.8% of the tokens are named entities spanning four categories: person, location, organization, and miscellaneous. The data were scraped from the Moroccan Dialect section of Wikipedia and processed and annotated using open-source libraries and tools. The data are useful for the Arabic natural language processing (NLP) community as they address the lack in dialectal Arabic annotated corpora. This dataset can be used to train and evaluate named entity recognition systems in dialectal and mixed Arabic.

**Specifications Table**

**Table:** 

Subject	Data science
Specific subject area	Identify named entities in textual data in the Moroccan Dialect
Type of data	Tabular
How the data were acquired	The data were programmatically scraped using the Wikipedia library for Python and cleaned and preprocessed using open-source tools
Data format	Raw Standardized
Description of data collection	The dataset was collected through automatic scraping and processing and manual annotation. Articles from the Moroccan Dialect section of Wikipedia were scraped using Selenium and the Wikipedia Python library. The resulting text files were then annotated using Doccano, a text annotation tool for a wide range of NLP tasks. The exported output files in JSONL format were then programmatically converted to a tabular CSV format following the BIO tagging scheme. Each row in the final dataset contains a token and its corresponding tag.
Data source location	The Web
Data accessibility	Public repository Repository name: Mendeley Data identification number: 10.17632/286sss4k9v.4 Direct URL to data: https://data.mendeley.com/datasets/286sss4k9v/4

**Value of the Data**


 

• DarNERcorp is the first annotated corpus for NER in the Moroccan dialect. It thus makes a valuable contribution to the available resources for dialectal Arabic (NLP).
• This dataset is useful for computer scientists and computational linguists interested in Arabic and its dialects.
• This dataset can be used to evaluate multi-dialectal Arabic transformer language models on the downstream task of NER.
· This dataset follows the BIO format and can thus be combined with other widely used corpora in standard and dialectal Arabic to train large models.
• Unlike Levantine, Gulf, or Egyptian dialects, there are very few datasets for the Moroccan dialect. Hence, this work aims to increase data availability and advance the state of the art of Moroccan Dialect NLP.

## 1 Objective

The aim behind the collection of this dataset is to obtain an accurately annotated corpus of NER of the Moroccan Dialect. Due to its small size, DarNERcorp can be used to fine-tune language models or be combined with existing corpora to train mixed Arabic models.

## 2 Data Description

After scraping, preprocessing, and annotating the data, the final dataset consists of 65,905 tokens, 13.8% of which represent named entities. There are four categories of named entities in the dataset: person (PER), location (LOC), organization (ORG), and miscellaneous (MISC). A named entity of type person generally consists of the name used to refer to a person. Location named entities refer to geographical locations, such entities include country and city names or well-known places. Organization named entities denote different types of institutions such as international organizations, associations, and companies. The miscellaneous category includes named entities that do not belong to the previously mentioned classes. Namely, in DarNERcorp, the miscellaneous category includes dates, nationalities, works of art, and names of events. The distribution of these named entities in the dataset is as follows:

• PER: 15.3%
• LOC: 38.1%
• ORG: 15.5%
• MISC: 31.1%

The dataset was tagged using the BIO tagging scheme, where BIO stands for “Beginning, Inside, Outside” [1]. The ‘O’ tag is assigned to tokens that do not represent a named entity. The beginning token of a named entity is given the tag ‘B’ followed by the category that it represents. For example, the first token of a location named entity is given the tag ‘B-LOC.’ The tokens that occur inside a named entity are given the tag ‘I’ followed by their specific category. For instance, the surname in an entity representing a person would have the tag ‘I-PER.’ Hence, the final dataset contains the following tag set: O, B-PER, I-PER, B-LOC, I-LOC, B-ORG, I-ORG, B-MISC, I-MISC. We assume that named entities are non-recursive and non-overlapping. DarNERcorp thus follows the same named entities taxonomy and tagging scheme as other widely used corpora in Arabic, such as ANERcorp [2]. This facilitates the combination of these datasets to train models for mixed Arabic.

The published dataset contains two folders: Code and Data. Code contains the Python scripts used to scrape data and convert the data from JSONL format to tabular format. The Data folder contains two sets: DarNERcorp_train and DarNERcorp_test. DarNERcorp_train contains 80% of the total dataset and DarNERcorp_test contains the remaining 20%. A description of the columns in the train and test sets as well as an example of a row in the datasets are provided in Tables 1 and 2.

**Table 1:** Description of columns in DarNERcorp dataset.

Column	Description
Token	A word, number, or punctuation mark representing one token
Tag	The tag assigned to the token according to the BIO tagging scheme

**Table 2:** Description of a row in DarNERcorp dataset.

Column	Value
Token	The word in Darija for “Morocco”
Tag	B-LOC

The number of instances of each category of named entities in the train and test sets is given in Table 3.

**Table 3:** Named entity statistics in DarNERcorp_train and DarNERcorp_test.

Category	DarNERcorp_train	DarNERcorp_test
LOC	2844	629
PER	1098	298
ORG	984	435
MISC	2264	578

## 3 Experimental Design, Materials and Methods

**Data scraping from Wikipedia**. The data used to create the corpus were scraped from the Darija section of Wikipedia. Wikipedia was chosen as the primary source of data because it contains many named entities and is generally error-free, unlike social media platforms. We used Selenium^1^ WebDriver to navigate through each Wikipedia article starting from the web page containing the list of links to all articles. We then employed the Wikipedia^2^ Python library to extract the text in each article. 5000 articles were scraped following this procedure and stored in text files. The full code of data scraping can be found on Mendeley^3^.

**Data annotation**. The text files obtained from the scraping phase were uploaded to Doccano^4^, an open-source text annotation tool. Doccano offers features for a wide range of NLP tasks, including sequence labeling, the category that NER belongs to. An instance of Doccano was hosted on Heroku^5^ to enable collaborative annotation. Two annotators, who are native speakers of the Moroccan dialect with previous experience in annotation for Darija NLP projects, were hired to annotate the articles. The annotators were trained about the task of NER and a markup file with annotator guidelines and exhaustive examples was uploaded to the annotation software. The inter-annotator agreement rate was 92.1%. Cases of disagreement were resolved with the authors to ensure data quality throughout the annotation process. Articles were annotated on a rolling basis, however those with very few named entities such as articles about dates and days of the week were avoided. After annotation was completed, the output files were exported from Doccano in JSONL format.

**Data formatting**. We developed a Python script to convert the exported data in JSONL format to a tabular format. Each line in the JSONL file consisted of an article stored in a dictionary with two main fields: the text field which contains a string representing the article, and the label field which is a list of lists that stores the starting and ending index of each named entity in the text along with its category. An example of an entry in the JSONL file is as follows:

{“text”: 6“”,

“label”: [[11, 16, ORG], [25, 30, LOC], [44, 50, LOC]]}

We used CAMeL tools, an Arabic natural language processing kit, to tokenize the text [3]. We then used the indices provided in the label field of the JSONL file to assign the appropriate tag to each token in the corpus. The first token in a named entity was tagged with a ‘B’ prefix and the following tokens with a ‘I’ prefix. The results were stored in a Pandas data frame and exported as a CSV file. The code used to make the conversion is available on Mendeley^7^.

**Reference results**. To test the usefulness of our data in training NER systems, we trained AraBERT^8^ model for token classification from HuggingFace^9^ on our train set and evaluated its performance on the test set. The model was trained on 5 epochs using a learning rate of 3e-5. The performance of the model on the test set, measured in terms of precision, recall, and F1-score is given in Table 4. AraBERT was used because of the lack of high-quality language models in the Moroccan dialect. The results are quite good and represent an important starting point for NER in the Moroccan dialect.

**Table 4:** Evaluation of reference model on DarNERcorp_test.

Category	Precision	Recall	F1-score
LOC	60.77	87.38	71.69
PER	60.12	63.64	61.83
ORG	42.73	51.05	46.52
MISC	50.52	49.37	49.93
Overall	53.25	67.37	59.48

## Ethics Statement

The data used to compile the dataset do not pose any ethical concerns as they were collected from Wikipedia and not a social media platform or other sensitive data sources. We did not need permission to use data from Wikipedia. We did not conduct human or animal studies in our work.

## CRediT authorship contribution statement

**Hanane Nour Moussa:** Software, Resources, Validation, Data curation, Writing – original draft. **Asmaa Mourhir:** Conceptualization, Writing – review & editing, Supervision, Project administration.

## Declaration of Competing Interest

The authors declare that they have no known competing financial interests or personal relationships that could have appeared to influence the work reported in this paper.

## Data Availability

DarNERcorp (Original data) (Mendeley Data).

## Footnotes

1 https://www.selenium.dev.

2 https://pypi.org/project/wikipedia.

3 https://data.mendeley.com/datasets/286sss4k9v/4/files/83b54468-f5fd-482d-96af-d7858e58026b.

4 https://github.com/doccano/doccano.

5 https://www.heroku.com.

6 The sentence in Darija to say “The headquarters of OPEC are in Vienna the capital of Austria”.

7 https://data.mendeley.com/datasets/286sss4k9v/4/files/daa51f9a-7ea8-4f53-93d8-19797b452ffb.

8 https://huggingface.co/aubmindlab/bert-base-arabert.

9 https://huggingface.co/.

## References

[1] L. Ramshaw and M. Marcus, “Text Chunking Using Transformation-Based Learning,” in *Proceedings of the Third ACL Workshop on Very Large Corpora*, 1995.

[2] Y. Benajiba, P. Rosso and J.M. Benedi Ruiz, "Anersys: an Arabic named entity recognition system based on maximum entropy," in Proceedings of the International Conference on Intelligent Text Processing and Computational Linguistics, Berlin, Heidelberg, 2007.

[3] O. Obeid, N. Zalmout, S. Khalifa, D. Taji, M. Oudah, B. Alhafni, G. Inoue, F. Eryani, A. Erdmann and N. Habash, "CAMeL tools: an open source python toolkit for Arabic natural language processing," in Proceedings of the Twelfth Language Resources and Evaluation Conference,Marseille, France. European Language Resources Association. pp. 7022–7032, 2020.

